# Presenting an efficient approach based on novel mapping for mortality prediction in intensive care unit cardiovascular patients

**DOI:** 10.1016/j.mex.2018.10.008

**Published:** 2018-10-09

**Authors:** Mohammad Karimi Moridani, Yashar Haghighi Bardineh

**Affiliations:** Department of Biomedical Engineering, Tehran Medical Sciences Branch, Islamic Azad University, Tehran, Iran

**Keywords:** ECG, electrocardiogram, HRV, heart rate variability, ICU, intensive care unit, RRI, the interval between R waves in the ECG, RP, risk plot, SCD, sudden cardiac death, Mortality prediction, ICU, Heart rate variability, Risk plot, Nonlinear Mapping

## Abstract

Intensive care unit (ICU) experienced and skillful people in this field should be employed because the equipment, facilities, and admitted patients have more special conditions than other departments. Our goal provides the best quality according to the condition each patient and prevent many unnecessary costs for preventive treatment.

In this paper, the proposed system will first receive the patient's vital signs, which are recorded by the ICU monitoring. After the necessary processing, in case of observing changes in the normal state, risk alarms are transmitted to the nursing station so that nurses become aware of this condition and take all equipment to return the patient to normal condition and prevent his death. The applied graph in this study examines patients at any moment and displays the patient's future condition in a schematic manner after precise analyses. In this algorithm, after calculating the R-R intervals in the electrocardiogram signal, RRIs are thrown into a risk plot (RP) by a projectile. Given the amount of projectile RRI, one of the stairs can host that amount. After a few moments by springs embedded under the stairs, the drain of RRIs is done by the kinetic energy stored in the springs towards the valley of life. If the accumulation of quantities in a stair is too much, the spring will not be able to project those RRIs. By examining this situation, we will introduce an index to determine the risk of death for all patients.

The results of this paper show that when a person is in normal condition, there is no density in a certain stair and the ball or the projected RRIs are not limited to a stair. In general, the results of this paper show that the lower amount of RRI dispersion in the RP leads to greater risk of entry into the death range and as this amount decrease, an immediate consideration is required.

In conclusion, if the precise prediction of the future condition of ICU patients is available to nurses and doctors, more facilities and equipment could be provided to save their lives.

• We focused on nonlinear methods with new aspects to extract mentioned dynamics.
• This method can reduce the number of ICU nurses and give the special facilities for high-risk patients.
• Our results confirm that it is possible to predict mortality based on the dynamical characteristics of HRV.

## Graphical abstract

**Figure:**
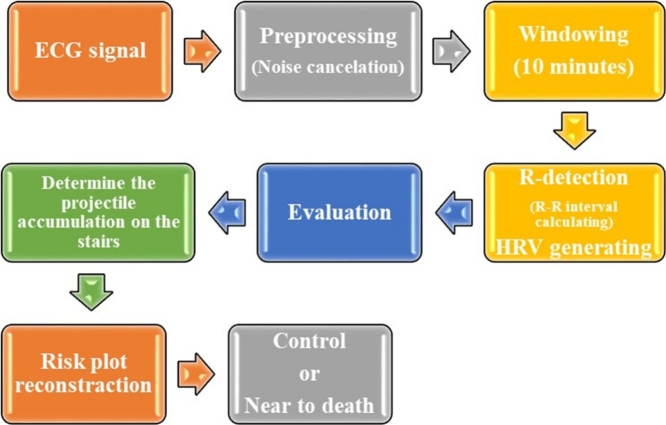

**Specifications table**

**Table:** 

**Subject Area**	*Engineering*
**More specific subject area:**	*Signal Processing*
**Method name**	*Nonlinear Mapping*
**Name and reference of original method**	Doi:10.3109/03091902.2016.1139201
**Resource availability**	*data, software*

## Method details

The intensive care unit (ICU) requires trained and experienced personnel and the devices and equipment that should be used for care, treatment and information monitoring of patients admitted to this section are much more specialized than other units [1]. Therefore, this unit of the hospital is one of the most expensive and important parts of all hospitals [2]. In addition to the high costs, this unit has also caused problems the most important of which is the lack of beds and the equipment needed in it [3]. As it is known, all ICU patients will not necessarily benefit from it and hospitalization will only result in more comfortable death in few cases [4].

So far, extensive research has been done to predict the mortality of cardiovascular patients. Here recent studies in this area will be reviewed and the results of the proposed methods will be reported by the researchers.

Cardiac arrhythmia can lead to irreparable dangers resulting in death. Each year, thousands of deaths occur in the United States, which are mostly related to adults [5,6]. The wave of cardiovascular diseases has affected the whole world and today it has become one of the causes of increased mortality. According to the latest figures, shown in India, about 30 million people in the country suffer from cardiovascular disease, and according to the studies conducted in this field, these figures are due to factors such as stress, unhealthy eating habits, lack of physical activity, lack of sleep, and alcohol and cigarette dependence [7]. Although deaths from heart disease have declined over the past 20 years [8], sudden cardiac death is still the cause of half of the deaths from cardiovascular diseases [9]. Early methods for predicting the mortality of heart patients include physician examination, examining genetic factors, medical tests, recording vital signals such as electrocardiogram (ECG) and their interpretation, stress test, magnetic resonance imaging (MRI) from the heart, and so on [10]. Data mining techniques also play a key role in the field of medicine, because they are used to discover, analyze and extract medical data using sophisticated algorithms [11,12]. Recently, researchers have focused on nonlinear methods because of the chaotic function of the heart rate variability (HRV) signal as important features can be extracted from it that can be effective in detecting and predicting mortality [13,14].

The first patient severity classification system called Acute Physiology and Chronic Health Evaluation (APACHE) appeared in 1981 at the University of George Washington. Newer versions of this tool, such as APACHE II and Simplified Acute Physiology Score (SAPS) were created in 1985 and 1993 [15,16] and are still commonly used. With the introduction of APACHE IV in 2006, it was suggested that old models should not be used for long periods of time because their false results can be increased. Finally, after many studies conducted to determine the validity of these tools, a new application of these tools, such as Sequential Organ Failure Assessment (SOFA), Mortality Prediction Model (MPM), Multiple Organ Dysfunction Score (MODS) and APACHE V, emerged [[17], [18], [19]].

A study was conducted in 2003 using APACHE II on 330 respiratory patients hospitalized in ICU. In this study, 287 patients survived and the remaining 43 patients died. The mean and standard deviation in the live and dead groups were 11.34 ± 6.75 and 23.09 ± 10 [20].

Another study by Safavi et al. [21] on APACHE II tool has compared this tool and other indicators to predict mortality. The results of this study indicate APACHE II's superiority in predicting mortality in patients with a sensitivity of 90% and accuracy of 81% [21].

Another study is conducted by Goyder et al on 301 patients with the death rate of 17.2%. The results of the use of the APACHE tool indicate that the mean and standard deviation in the surviving and deceased patients were 12.94 ± 7.43 and 28.19 ± 10.43 [22].

Shen et al. [23] presented a personal cardiac system to predict sudden cardiac death two minutes before the onset of it. In their research, they used the characteristics of the ECG and the HRV signals of 20 healthy people and 23 patients with sudden cardiac death (SCD) and using the HRV short-term and wavelet analysis they differentiated between healthy subjects and SCD patients by 87.5%. Finally, these features were used in artificial neural networks. The accuracy of SCD prediction was 67.44%, 58.14%, and 55.81%, using the least square, decision-based neural network, and back propagation neural network methods. Voss et al. [24] proposed a method using time and frequency characteristics of ECG and HRV signals of 35 healthy subjects and 26 cardiac patients after myocardial infarction (divided into low risk and high-risk groups). They used nonlinear and renormalized entropy methods. They classified the healthy people and high-risk patients with 96% accurately by nonlinear methods. Using time and frequency domain parameters the accuracy of the results was less than 90%. They also managed to create 100% accuracy with a combination of different features in multiple domains and stepwise discriminant function.

La Rovere et al. [25] used time and frequency domain parameters as well as features extracted from the HRV signal to predict SCD. Their results indicated that short-term low-frequency power of HRV signal during controlled breathing is effective in predicting sudden cardiac death in patients with chronic heart failure.

Bilgin et al. [26] presented a new method that has been used to diagnose ventricular tachyarrhythmia. In their study, they showed that some subbands such as low frequency (LF) and high frequency (HF) have lower energy values than other subbands for ventricular tachyarrhythmia. In another study by Bilgin et al. [27] the very low frequency (VLF) subband was described as the dominant subband for the evaluation of ventricular tachyarrhythmia.

George et al. [28] used non-linear methods (Poincare plot, Detrended Fluctuation Analysis (DFA), Hurst index and approximate entropy), geometric methods (triangular index), frequency analysis methods (power in LF and HF bands) and statistical methods (NN (R—R) intervals which was greater than 50 ms (pNN50), Root Mean Square of the Successive Differences (RMSSD), Standard Deviation of Successive Differences (SDSD), Standard deviation of the NN intervals (SDNN)) in HRV signal analysis. They used the HRV signal for 40 patients with heart failure (high and low risk). The classification accuracy using the *S*upport Vector Machine (SVM) with Radial Basis Function (RBF) core and random forest classifiers were 85% and 87.5% respectively. The linear and nonlinear features to classify cardiovascular heart disease was presented by Moridani et al. [2,29].

The paper is arranged as follows: the second section examines the data used and the method presented in this article. The third section is dedicated to the results using the method presented in this paper and the discussion and conclusion are also presented in the fourth section.

## Methods

### Data collection

The number of cardiovascular patients needed to predict their future status was determined as 50 subjects (25 males, 25 females) aged 45–65 years based on the results of multiple analyses and validation of the results. The sampling frequency was 256 Hz and the data recording time varied between 8 and 45 h. The power-line interference is a noise created by the electrical devices. A 50 or 60 Hz notch filter was typically done as filter to avoid power-line interference. The point that was considered in the patient data record was the date to start recording. Given that ICU patients are considered as patients with poor health conditions, it is better to record data from the beginning or at the moment of the patient's admission to this unit to make a good prediction of their future status. Nurses and medical staff also monitored the status of the studied patients from the moment they entered the unit until the moment of death and recorded all the information and taken actions. We have focused on cardiovascular patient and our patients did not have a history of heart problems and diabetes. In this paper, people under the age group of 18 years old as well as those who were hospitalized due to trauma and burns in the heart ICU were excluded from the study population. Another point that is important in recording data from patients is the recording time. Considering that patients entering the ICU are among critically ill ones, it is better to record data from the very beginning or in other words at the moment of the arrival each patient in this unit, in order to predict the correctness of their future trend. Regarding the fact that it is very difficult and sometimes impossible to record the ill patients in the ICU, however, a total of fifty records were made for the purpose of this study. More details about the data are summarized in Table 1.

**Table 1:** Characteristics of studied cardiovascular patients.

Characteristic	All subjects (n = 50)
Mean (Standard deviation) age (years)	59.7 (8.2)
Sex (%Men)	50
Mean (Standard deviation) height (cm)	169.2 (4.2)
Mean (Standard deviation) weight (kg)	81.2 (3.8)
Length of stay (h)	8–45

### The proposed algorithm

**Fig. 1:**
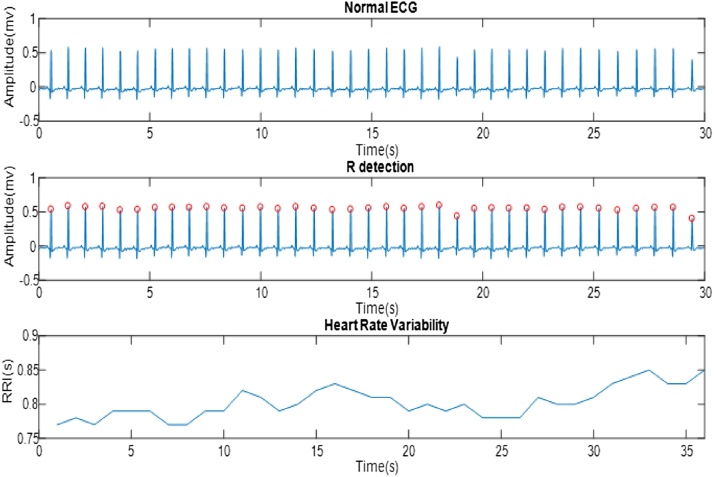
The image of the ECG signal (above signal) along with the peaks (middle image) and the HRV signal (lower image).

The use and analysis of the ECG signal are one of the methods for examining and diagnosing cardiovascular diseases. There is a series of QRS waves in each ECG signal that consist of three waves called Q, R, and S. The distance between the R wave per beat and the next R wave is referred to as RR interval. The distance between these beats varies depending on the individual's condition at different times. The signal caused by RR interval over time is called the HRV [10]. Fig. 1 shows the image of the ECG signal with the detection of R peaks and the production of the HRV signal.

**Fig. 2:**
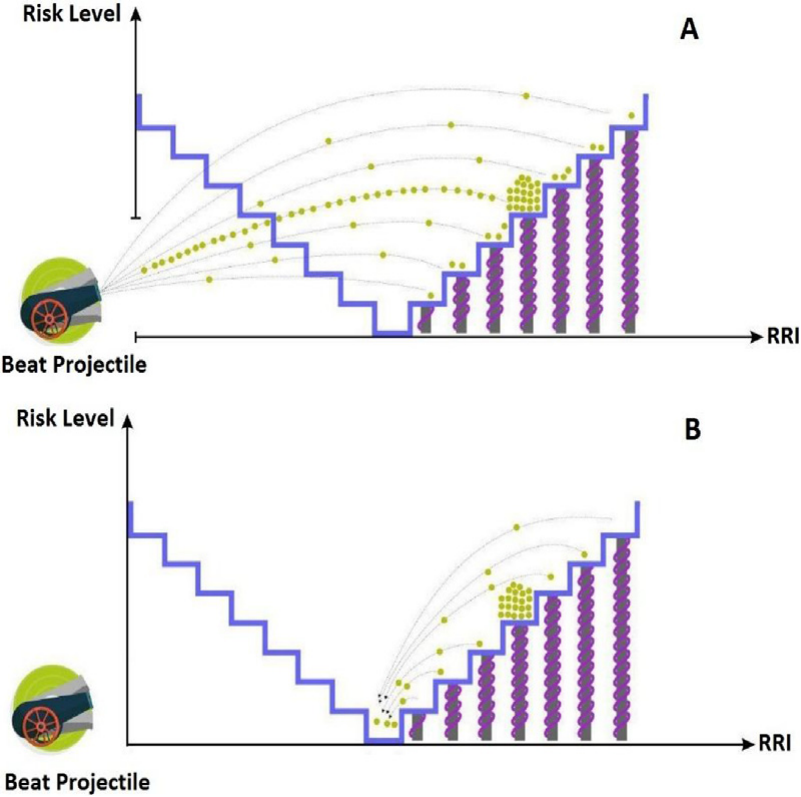
HRV signal dynamic representation at moments close to death by mapping the risk.

In this paper, after admitting the patient and recording the ECG signal by ICU monitoring, this signal is preprocessed. In the preprocessing stage, the signal noise was eliminated by the Daubechies method using the wavelet method [30] to further enhance the success of other processing processes in order to produce the desired result. Also the power-line interference was removed using notch filter. Since the ECG signal is similar to the normal signal in many diseases and important diagnostic information cannot be extracted from this signal [31], in this paper, the HRV signal is used to predict the patient's future condition in admission hours in this unit up to moments before death. Regarding the intrinsic nature of the HRV, which is considered as a chaotic signal, nonlinear processing is used to extract information, analysis, and evaluation. Given the hidden important information in time signals, the deformation of these signals and the creation of a mapping of these signals can be very useful in extracting specific information and helping to better predict the future condition of patients [3,4]. The proposed mapping in this paper has more information than any other mapping in the field of diagnosis and prediction in addition to the dynamics. Fig. 2 shows an image of this mapping that consists of two axes; the horizontal axis represents the RR interval and the vertical axis shows the person's risk appetite. This map contains two horizontal and vertical axes, which is called the risk plot (RP) according to its design. The horizontal axis represents the R-R Interval and the vertical axis shows the person's risk level. This plot contains other parts such as stairs, projectiles and a number of balls. There are stairs in the plot that show the landing point of the projected RRIs. In this method, after calculating the R-R Intervals, the IRRs are projected into the RP by a projectile. Given that the projected RRI value, it will land on one of the designed stairs. After a few moments, the RRIs are discharged into the life valley by the kinetic energy stored in the springs embedded below the stairs. If the accumulation of quantities in one of the stairs is too high, the spring will not be able to throw those RRIs. By examining this situation for all patients, an indicator is introduced to determine the patient's risk level at near to death moments.

In Fig. 2A the RRIs are projected to a risk plate by a beat projectile. Due to the amount of projected RRI, one of the stairs will host these values. After moments with springs embedded below the stairs, the RRI drainage action is performed with a constant force for each stair towards the valley of life. If the accumulation of quantities in a stair is too high, the spring cannot project those RRIs into the valley of life. After assessing the accumulation of RRIs on the stairs and the density of RRIs produced in the valley of life, the level of risk appetite for entering the death range is determined.

**Fig. 3:**
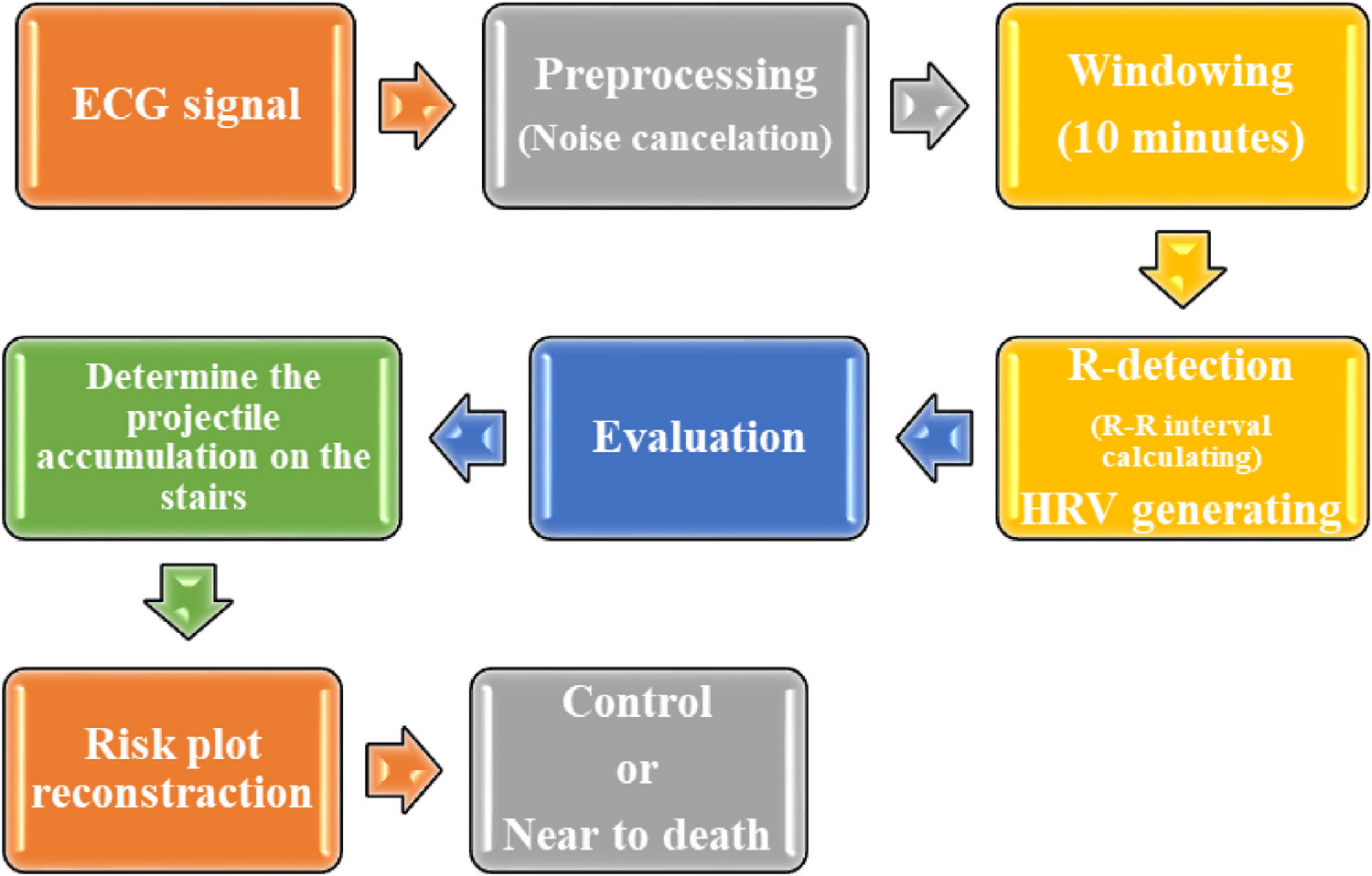
Block diagram for predicting the person's condition.

In this paper, with the help of vital signal processing techniques for the patient, the deaths of cardiovascular patients admitted to the cardiac intensive care unit are predicted with the aim of warning nurses and doctors of the unit to save lives of patients. For this purpose, the risk plot has been designed whose horizontal axis represents the RR Interval, the vertical axis is the level of the risk for each person, and stairs embedded inside the plot, each showing the landing site of projectile RRI. In this algorithm, after calculating the heart RR intervals, RRIs are thrown into an RP by a projectile. Given the amount of projectile RRI, one of the stairs can host that amount. After a few moments by springs embedded under the stairs, the drain of RRIs is done by the kinetic energy stored in the springs towards the valley of life. If the accumulation of quantities in a stair is too much, the spring will not be able to project those RRIs. By examining this situation, we will introduce an index to determine the risk of death for all patients. Fig. 3 shows a block diagram for proposed algorithm in this paper.

## Results

The results of this study clearly show that when a person is in a natural state, the springs can project the RRIs from the stairs by reducing the density on the stairs towards the valley of life. But as the person approaches the death, the projectile accumulation on one or two stairs will be excessive (due to HRV reduction [1,2,27]). If the accumulation of projectiles some stairs is excessive, the springs will not be able to throw RRIs into the valley of life and there will be a critical or near-death situation. The results of this study showed that as the dispersion rate in the RP is lower, the risk of entry into the death range will be greater. Fig. 2 shows a particular case where a person is approaching death and eventually leading to his death. In this case, the person's HRV reduces compared to the moments away from death. So Beat Projectile has projected the RRIs on certain stairs much more than other stairs which means the increased weight of that stair along the projectile. This causes the energy stored in the spring to lack the ability to project RRIs into the Valley of Life which is clearly shown in Fig. 2B. HRV in cardiovascular patients, which eventually resulted in their death, shows a significant decrease in mean and standard deviation of HRV values at moments close to death which is reduced from 7.18 ± 0.75 to 4.75 ± 0.21.

## Discussion and conclusion

Doctors and specialists of specialized care units should have a perspective of the final condition of their patients along with careful examination of the progression of the disease and the treatment of patients in order to utilize the capacity of this department in an optimal manner and specify those who need more attendance or care in the ICU [3,4]. This will make it necessary for the unit to be equipped with devices equipped with a very high level of alert and smartly sensitive to vital signs so that when the patient’s life is at risk, the device would alarm correctly and save his life. Therefore, the correct prediction of the future condition of patients can be effective in the timely treatment and reduce mortality in this unit.

Accurate classification of the diseases and accurate prediction of the outcome of patients can optimize the use of ICU beds, reduce the financial burden on the individuals and insurance companies and decrease mortality in the community by reducing unnecessary monitoring. Nowadays, the use of new and advanced techniques and technologies has made it possible to anticipate and treat most diseases in ICU resulting in the longer survival of patients [29]. Given that this paper is focused on the patient's heart rate signal, recording other parameters of the patients should not be ignored. Therefore, it is recommended to use other patient data such as systolic and diastolic blood pressure and even respiratory rate to evaluate and correct the algorithm. On the other hand, with the help and utilization of the proposed risk mapping in this article, one can analyze how the patient entered the risk of death range and, with the warning of death risk, and save the patient from death by medical and therapeutic staff. Another advantage of the proposed system of this paper in the prognosis of cardiovascular deaths in intensive care units is the production of an online software package and its application in remote medicine for the exchange of information among various medical teams and the adoption of therapeutic approaches. Using such a system in each special care unit can be a criterion for determining the standardized department and compare the quality of services with other departments of the hospital as well as comparing them with the global standard.

## Conflict of interest

The author who name is listed in this manuscript has no affiliation with or involvement in any organization or entity with any financial interest or nonfinancial interest in the subject matter or materials discussed in this manuscript. The author has also declared that no conflict of interest exists.

## Ethical considerations

Ethical issues (Including plagiarism, informed consent, misconduct, data fabrication and/or falsification, double publication and/or submission, redundancy, etc.) have been completely observed by the authors.

## Ethical statement

Additional informed consent was obtained from all patients for which identifying information is included in this article.
